# Ferroptosis: a dual-edged sword in tumour growth

**DOI:** 10.3389/fphar.2023.1330910

**Published:** 2024-01-11

**Authors:** Xiangye Zhao, Xiaoning Li, Yinghui Xu

**Affiliations:** Cancer Center, The First Hospital of Jilin University, Changchun, Jilin, China

**Keywords:** ferroptosis, tumour, iron, metabolism, antitumour therapy

## Abstract

Ferroptosis, a recently identified form of non-apoptotic cell death, is distinguished by its dependence on iron-triggered lipid peroxidation and accumulation of iron. It has been linked to various disorders, including the development of tumours. Interestingly, ferroptosis appears to exhibit a dual role in the context of tumour growth. This article provides a thorough exploration of the inherent ambivalence within ferroptosis, encompassing both its facilitation and inhibition of tumorous proliferation. It examines potential therapeutic targets associated with ferroptosis, the susceptibility of cancerous cells to ferroptosis, strategies to enhance the efficacy of existing cancer treatments, the interaction between ferroptosis and the immune response to tumours, and the fundamental mechanisms governing ferroptosis-induced tumour progression. A comprehensive understanding of how ferroptosis contributes to tumour biology and the strategic management of its dual nature are crucial for maximizing its therapeutic potential.

## 1 Introduction

Cell death plays a crucial role in maintaining tissue balance and controlling the unregulated growth of tumour cells (Fuchs and Steller, 2011). However, tumour cells have evolved mechanisms to evade cell death regulation, promoting unchecked cell replication. Ferroptosis, a unique form of non-apoptotic cell death characterised by lipid peroxidation and unstable iron buildup, differs in morphology, physiology, and biochemistry from classical programmed cell death (Dixon et al., 2012; Friedmann Angeli et al., 2014; Stockwell et al., 2017; Hassannia et al., 2019). An increasing body of evidence implicates ferroptosis in the development of various diseases, including the onset and progression of tumours (Tang et al., 2021).

Currently, ferroptosis has emerged as a significant focus in oncology research. Most studies suggest a beneficial role in restraining tumour growth through interactions between ferroptosis and tumours (Table 1), highlighting its potential as a therapeutic target in oncology. Tumour cells can bypass ferroptosis to promote their own growth by employing defense mechanisms, such as activating System Xc-, boosting glutathione peroxidase 4 (GPX4) activity, and altering glutathione (GSH) metabolism (Dixon et al., 2012). Disrupting or eliminating these mechanisms can trigger ferroptosis and hinder tumour expansion. Additionally, regulating lipid metabolism and iron metabolism pathways can induce ferroptosis, thereby inhibiting tumour growth (Martinez-Outschoorn et al., 2017; Wolpaw and Dang, 2018; Sang et al., 2019; Zou et al., 2020; Lei et al., 2022). Ferroptosis inducers presents a promising approach to curbing tumour growth. Furthermore, combining ferroptosis with chemotherapy, radiotherapy, targeted therapy, or immunotherapy shows potential to enhance antitumour effectiveness and overcome drug resistance (Yamaguchi et al., 2013; Yu et al., 2015; Wang et al., 2019a; Ye et al., 2020) (Table 3). Consequently, ferroptosis holds the potential to reshape tumour treatment strategies and improve clinical outcomes.

**TABLE 1:** Inhibitory effect of ferroptosis on tumour growth by regulating metabolisms.

Mechanism category		Mode of functioning	Function	*In vitro*/vivo	Model	Tumour type	References
Regulate lipid metabolism		Chemerin	Downregulate peroxidized PUFAs, evade ferroptosis, support RCC growth	*In vitro*	ccRCC model systems	RCC	Tan et al. (2021)
		KRAS mutation	Increase the expression of ACSL3, promote MUFA-PL biosynthesis, ferroptosis resistance, facilitate lung cancer progression	*In vitro* and vivo	A549 and H460 NSCLC cells; *KrasG12D* (*tet-op-KrasG12D*) mice	Lung cancer	Friedmann Angeli et al. (2014), Padanad et al. (2016)
		Mesenchymal tumour cells	Overexpression of ELOVL5 and FADS1, involve PUFAs synthesis, render cancer cells susceptible to ferroptosis	*In vitro*	Mesenchymal-type GCs (including Hs746T, SNU-484, SNU-668, YCC-16, and SNU-216 cells)	Mesenchymal GC cell	Lee et al. (2020)
Regulate amino acid metabolism	GPX4	RSL3	Inactivate GPX4, induce ferroptosis, inhibit tumour growth	*In vivo*	Xenograft mouse model of BJeLR cell origin	Fibroblastic tumour	Fuchs and Steller (2011)
		FIN56	Deplete GPX4 protein, block coenzyme Q10 production, induce ferroptosis	*In vitro*	HT-1080 fibrosarcoma cells and BJeLR cells	Fibroblastic tumour	Badgley et al. (2020)
	GSH	Kras/TP53 mutation	Deplete cystine or cysteine, induce ferroptosis, inhibit tumour growth	*In vitro* and vivo	Human PDAC cell lines and KPC mice	Kras/TP53-driven PDACs	Badgley et al. (2020)
		Sulfasalazine	Inhibits System Xc- and diminish cellular glutathione, induce ferroptosis, inhibit pancreatic cancer growth	*In vitro* and vivo	Human pancreatic cancer cell lines MIA PaCa-2 and PANC-1; mice bearing actively growing MIA PaCa-2 and PANC-1 subcutaneous xenografts	Pancreatic cancer	Lo et al. (2010)
	SLC7A11	Overexpression of SLC7A11	Augment cystine uptake and GSH synthesis, inhibit ferroptosis, promote tumour growth	*In vitro*	Human PDAC cell lines	PDACs	Badgley et al. (2020)
		SLC7A11, KRAS-mutant	Mediate cystine uptake, decrease ROS production, inhibit ferroptosis, promote lung adenocarcinoma proliferation and migration	*In vitro* and vivo	NSCLC A549 cells; lung cell lines (HPNE and HPNE/KRAS; H522 and H522/KRAS); LSL-KrasG12D mouse	KRAS-mutant LUAD	Hu et al. (2020)
		siRNA	Downregulation of SLC7A11, induce ROS accumulation, promoted ferroptosis, inhibit lung cancer cell proliferation	*In vitro*	A549 cell	Lung cancer	Huang et al. (2018)
		XAV939	Downregulate SLC7A11 through lncRNA, induce ferroptosis, inhibit NSCLC	*In vitro*	NCI-H1299 NSCLC cell line	NSCLC	Yu et al. (2019)
Regulate iron metabolism		Artemisinin	Absorb and release iron, heighten their susceptibility to ferroptosis, inhibit tumour growth	*In vitro* and vivo	Mouse embryonic fibroblasts and human osteosarcoma HT1080 cells; athymic nude *Foxn1nu/Foxn1* mice of GPX4 knockout in H292 cells	Lung cancer	Chen et al. (2020)
		Iron metabolism	Increase iron uptake, decrease iron efflux pump FPN, promote the onset of ferroptosis, inhibit tumour growth	*In vitro* and vivo	HGSOC tumour initiating cells; mice inoculated IP with FPN-tet-on FTt cells	HGSOC	Basuli et al. (2017)
		Iron oxide nanoparticles	Release intracellular iron, increase iron and ROS production, induce ferroptosis and hinder tumour growth	*In vitro* and vivo	A2780 and ACP cells; mice of H22 liver cancer model	Ovarian cancer; liver cancer	Ma et al. (2017)

PUFAs, polyunsaturated fatty acids; RCC, renal cell carcinoma; ACSL3, Acyl-coenzyme A synthetase long chain family member 3; MUFA-PL, Monounsaturated fatty acids-phospholipids; NSCLC, non-small cell lung cancer; ELOVL5, elongation of very long-chain fatty acid protein 5; FADS1, fatty acid desaturase 1; GC, gastric cancer; GPX4, glutathione Peroxidase 4; PDAC, pancreatic ductal adenocarcinomas; GSH, glutathione; LUAD, lung adenocarcinoma; HGSOC, high-grade serous ovarian cancer; EGFR, epidermal growth factor receptor; FPN, ferroportin; ROS, reactive oxygen species.

However, it’s important to note that ferroptosis can also have a negative impact on promoting tumour growth (Table 2). Through various pathways, such as ferroptosis metabolic pathways (Dai et al., 2020), inflammation-related pathways (Tang et al., 2021; Li and Li, 2020), antigen presentation process (Legrand et al., 2019), and the modulation of immune cell function (Wang et al., 2020; Luo et al., 2021), ferroptosis has been identified as a promoter of tumour growth. This article offers a comprehensive review of ferroptosis’s dual role in both promoting and inhibiting tumours, laying a theoretical foundation for further research into ferroptosis in tumour treatment. A thorough understanding of this duality allows for maximizing the clinical effectiveness of ferroptosis-based treatments while minimizing potential adverse effects.

**TABLE 2:** Promotional effect of ferroptosis on tumour growth.

Mechanism category	Pathway	Mechanisms	Effect	References
Regulate ferroptosis metabolic pathways	Lipid metabolism pathway	High expression of ACSL4, induce ferroptosis, promote fibrosis and hepatocellular cell formation	Promote tumour growth in hepatocellular carcinoma	Tsurusaki et al. (2019), Li et al. (2020a), Luo et al. (2020), Ndiaye et al. (2020), Qi et al. (2020)
	8-OHdG-TMEM173 pathway	GPX4 deletion or an iron-rich diet; induce ferroptosis, activate and migrate macrophages	Promote the development of Kras-driven pancreatic cancer	Cooke et al. (2003), Dai et al. (2020)
Trigger inflammation-related pathways	COX-2/PGE2 pathway	LPS induce ferroptosis, impact on NK cell activity, impede cDC1s and Immunosuppression	Evade the immune system, tumour immunotherapy resistance	Yang et al. (2014), Li and Li (2020), Tang et al. (2021)
	IFN-γ secretion by T cells	Upregulate PD-L1 expression, trigger IFN-γ-related adaptive immune resistance, evade the immune response	Promote tumour progression	Dorand et al. (2016), Tang et al. (2019), Zhang et al. (2019), Li et al. (2021)
	CD36-mediated ferroptosis	T cells take up fatty acids through CD36, induce ferroptosis, diminish the production of cytotoxic cytokines	Impair the antitumour activity of CD8 (+) T cells, promote tumour growth	Su et al. (2020), Wang et al. (2020)
Cancer cells dying from ferroptosis compromise antitumour immune responses	Tumour-associated antigen presentation process	Hinder DCs maturation, phagocytosis, influence neighbouring tumour cells to immunogenic death	Reduce antitumour immunity	Wiernicki et al. (2022)
	PMN-MDSCs	PMN-MDSCs undergo ferroptosis, promote the secretion of immunosuppressive molecules, Inhibit T cell activity	Promote tumour growth	Kim et al. (2022)

LPS, lipopolysaccharide; DCs, dendritic cells; 8-OHdG, 8-hydroxy-2′-deoxyguanosine; IFN-γ, interferon-γ; PD-L1, programmed death ligand-1; PMN-MDSCs, Polymorphonuclear myeloid derived suppressor cells; COX-2, cyclooxygenase-2; PGE2, Prostaglandin E2.

## 2 The mechanism of ferroptosis

**FIGURE 1:**
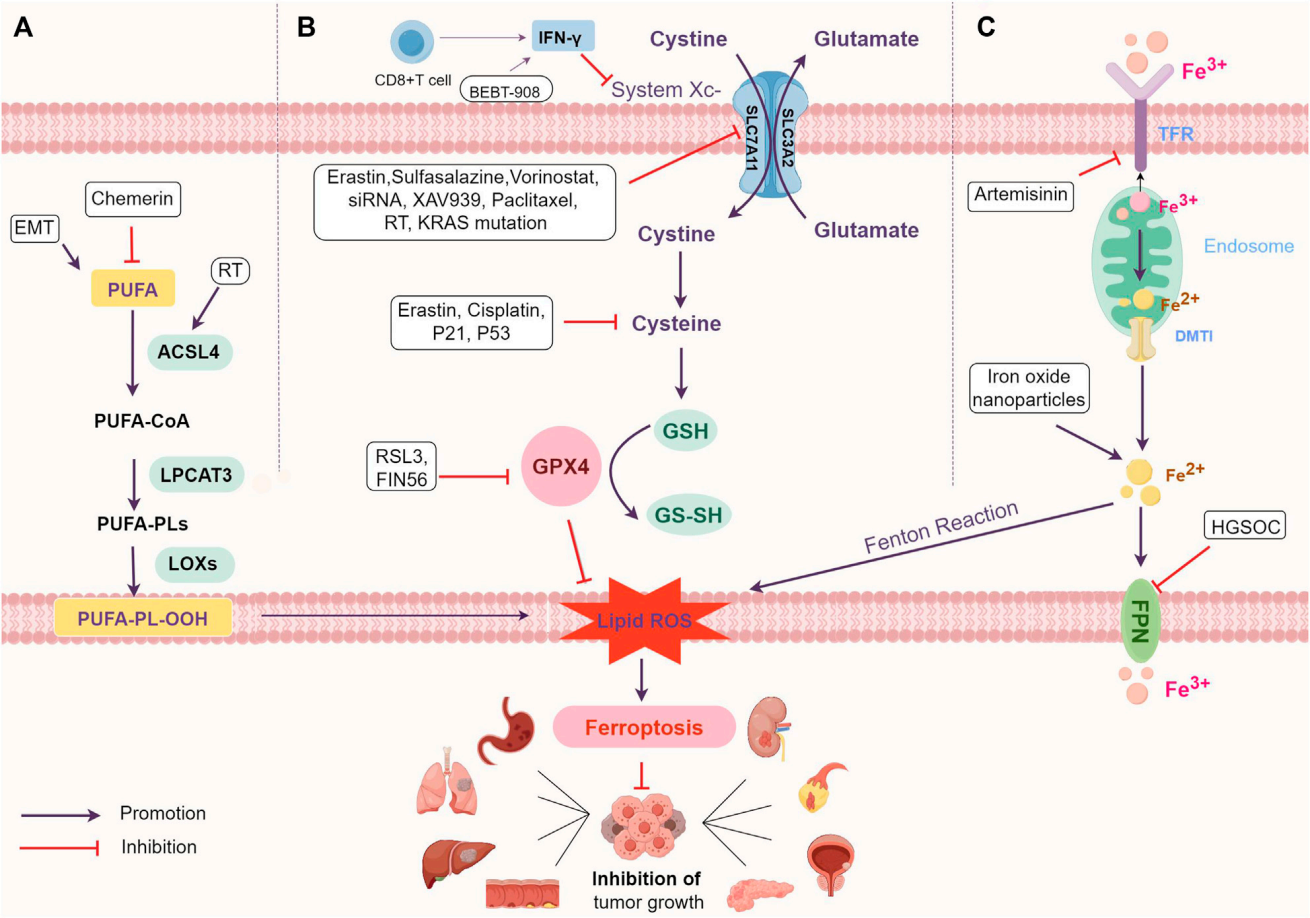
The mechanism of ferroptosis and main regulatory mechanism. **(A)** Regulate lipid metabolism pathways. **(B)** Interfere with amino acid metabolism pathways. **(C)** Influence iron metabolism pathways. Abbreviations: PUFAs, polyunsaturated fatty acids; PUFA-PLs, PUFA-containing phospholipids; RT, radiation therapy; ACSL4, Acyl-coenzyme A synthetase long chain family member 4; LPCAT3, Lysophosphatidylcholine acyltransferase 3; LOXs, lipoxygenase; EMT, epithelial-mesenchymal transition; ROS, reactive oxygen species; IFN-γ, interferon-γ; GSH, glutathione; GPX4, glutathione Peroxidase 4; TFR, transferrin; DMT1, divalent metal ion transporter protein-1; FPN, ferroportin; HGSOC, high-grade serous ovarian cancer.

Ferroptosis is an iron-dependent type of programmed cell death caused by excessive polyunsaturated fatty acids (PUFAs). PUFAs are essential components of cell membrane phospholipid layers, significantly influencing membrane structure, fluidity, and permeability (Luo et al., 2021). The main mechanism behind ferroptosis is that when the balance between cellular oxidation and the antioxidant system is disrupted (Kuang et al., 2020), PUFAs in the cell membrane undergo oxidation, forming hydroxyl radicals catalyzed by Fe2+ or ester oxygenase (Figure 1). This process creates lipid peroxides, leading to cellular ferroptosis (Yang and Stockwell, 2016; Yang et al., 2016; Stockwell et al., 2017). The products of lipid peroxidation in cell membranes act as a source of reactive oxygen species (ROS), triggering increased cellular oxidative stress that damages DNA, proteins, or lipids, ultimately resulting in cellular ferroptosis (Trachootham et al., 2009; Reczek and Chandel, 2018).

## 3 Inhibitory effect of ferroptosis on tumour growth

Ferroptosis can inhibit tumour growth. The inhibitory effect of ferroptosis on tumour growth is discussed in terms of regulating lipid metabolism, amino acid metabolism, and iron metabolism. It has been reported that inhibition of ferroptosis can also promote tumour growth.

### 3.1 Inhibition of ferroptosis through regulating lipid metabolism thereby promoting tumour growth

Lipid metabolism is closely related to ferroptosis. Lipid peroxidation is a free radical-driven reaction that primarily affects unsaturated fatty acids in cell membranes (Tang et al., 2021). Acyl-coenzyme A synthetase long chain family member 4 (ACSL4) and Lysophosphatidylcholine acyltransferase 3 (LPCAT3) are key regulators of PUFA-PLs synthesis. Phospholipase A2 (PLA2) cleaves PUFAs into free PUFAs and lysophospholipids (Tang et al., 2021). ACSL4 catalyzes the attachment of free PUFAs to coenzyme A to generate PUFA-CoAs, which are re-esterified and incorporated into phospholipids (PLs) by LPCAT3 to form PUFA-containing phospholipids (PUFA-PLs) (Lei et al., 2022; Doll et al., 2017; Dixon et al., 2015). Due to the presence of a bis-allylic moieties of PUFAs, PUFA-PLs are especially susceptible to peroxidation (Conrad and Pratt, 2019).

The downregulation of PUFAs in tumour cells is associated with ferroptosis evasion and the promotion of tumour growth (Lei et al., 2022) (Figure 1; Table 1). For instance, in renal cell carcinoma (RCC), reducing peroxidized PUFAs through the adipokine chemerin allows tumour cells to avoid ferroptosis and supports RCC growth (Tan et al., 2021). KRAS mutations in lung cancer also increase the expression of Acyl-coenzyme A synthetase long chain family member 3 (ACSL3) to reprogram lipid metabolism, promote Monounsaturated fatty acids-phospholipids (MUFA-PL) biosynthesis and ferroptosis resistance, and facilitate lung cancer progression (Friedmann Angeli et al., 2014; Padanad et al., 2016).

In human tumour cell lines, cells in a mesenchymal-like state show selective susceptibility to ferroptosis (Sang et al., 2019). Research indicates that mesenchymal tumour cells exhibit higher enzyme activity, promoting PUFAs synthesis and lipid peroxide production, ultimately leading to ferroptosis occurrence (Viswanathan et al., 2017; Xu et al., 2019) (Figure 1). Specific overexpression of elongation of very long-chain fatty acid protein 5 (ELOVL5) and fatty acid desaturase 1 (FADS1) in mesenchymal gastric cancer cells, both involved in PUFAs synthesis, makes cancer cells particularly susceptible to ferroptosis (Lee et al., 2020) (Table 1).

### 3.2 Escaping ferroptosis by interfering with the antioxidant system and affecting amino acid metabolism contributes to tumour growth

Ferroptosis is associated with disruption of the antioxidant system and amino acid metabolism (Figure 1). GSH-GPX4 is involved in the intracellular antioxidant system and is a key factor influencing the onset of ferroptosis. GPX4, the only member of the GPX protein family capable of converting phospholipid hydroperoxides into phosphatidyl alcohols, prevents lipid peroxidation, thus restraining ferroptosis and supporting tumour growth (Ursini et al., 1982; Brigelius-Flohé and Maiorino, 2013; Seibt et al., 2019; Brigelius-Flohé and Flohé, 2020). GSH, a co-factor for GPX4, is synthesized from glycine, glutamate, and cysteine, with cysteine being the rate-limiting precursor (Forman et al., 2009; Koppula et al., 2018; Friedmann Angeli et al., 2019).

Cysteine/glutathione antiporter, also known as System Xc-, is an important intracellular antioxidant element. System Xc-is a transmembrane protein, consisting of SLC7A11 and SLC3A2, responsible for the exchange of extracellular cystine with intracellular glutamate (Bannai, 1986; Conrad and Sato, 2012). SLC7A11 mediates cystine/glutamate antotransporter protein activity and SLC3A2 maintains SLC7A11 protein stability (Bannai, 1986; Sato et al., 1999; Conrad and Sato, 2012; Koppula et al., 2018). Therefore, inhibition of System Xc—leads to an imbalance of the antioxidant system thereby causing ferroptosis.

The SLC7A11-GSH-GPX4 system plays a crucial role as the main defense against ferroptosis in tumours (Dixon et al., 2012; Friedmann Angeli et al., 2014; Stockwell et al., 2017; Hassannia et al., 2019). GPX4 is a central control factor of ferroptosis, and intracellular GSH content directly affects GPX4 activity (Maiorino et al., 2018). Ferroptosis inducers have demonstrated efficacy in tumour cells by directly binding to and inhibiting GPX4 (Table 1). The ferroptosis activator RSL3, an inhibitor of the antioxidant system, directly inactivates GPX4 and inhibits tumour growth in a xenograft mouse model of BJeLR cell origin (Fuchs and Steller, 2011). FIN56, induces ferroptosis in HT1080 cells by depleting GPX4 protein as well as activating farnesyl-diphosphate farnesyltransferase 1 (FDFT1/SQS) to block coenzyme Q10 production (Shimada et al., 2016). Kras/TP53-driven pancreatic tumours induce ferroptosis and inhibit tumour growth by depleting cystine or cysteine through cyst (e) inase (Badgley et al., 2020). Sulfasalazine inhibits System Xc- and diminishes cellular glutathione, leading to the excessive buildup of lipid peroxides in tumour cells, inducing ferroptosis. This demonstrates an antitumour effect in pancreatic cancer (Lo et al., 2010).

In pancreatic ductal adenocarcinomas (PDACs), overexpressing SLC7A11 inhibits ferroptosis by increasing cystine uptake and GSH production, promoting tumour growth (Badgley et al., 2020). In patients with KRAS-mutant lung adenocarcinoma (LUAD), SLC7A11 mediates cystine uptake, decreases ROS production and thus promotes lung adenocarcinoma proliferation and migration (Hu et al., 2020; Liu et al., 2020; Chen et al., 2021; Lou et al., 2021). These suggest that SLC7A11 overexpression is positively associated with tumour progression. In contrast, downregulation of SLC7A11 gene expression by siRNA induced ROS accumulation, promoted ferroptosis and inhibited A549 cell proliferation (Huang et al., 2018). XAV939 induces ferroptosis and inhibits non-small cell lung cancer (NSCLC) by downregulating SLC7A11 through long non-coding RNA (lncRNA) (Yu et al., 2019).

### 3.3 Inhibiting tumour growth by affecting iron metabolism pathways to induce ferroptosis

Iron metabolism is a necessary process for ferroptosis. Iron overload induces ferroptosis through the Fenton reaction, which generates a large number of hydroxyl radicals and triggers a strong oxidative stress response that produces a large number of ROS (Conrad and Pratt, 2019). Transferrin (TFR) and divalent metal ion transporter protein-1 (DMT1) take up extracellular iron, and ferroportin (FPN) transfers intracellular iron to the outside of the cell (Figure 1). These proteins collaborate to maintain intracellular iron homeostasis (Seiler et al., 2008; Mandal et al., 2010). Iron is also essential for participation in lipid peroxidation, and lipoxygenase (LOXs) and cytochrome P450 oxidoreductase (PORs) require iron for catalysis (Jiang et al., 2021a).

Tumour cells show an increased demand for iron and display heightened oxidative metabolic processes compared to non-malignant cells (Martinez-Outschoorn et al., 2017; Wolpaw and Dang, 2018; Zou et al., 2020). The level of intracellular iron impacts sensitivity to ferroptosis. Elevated intracellular iron in tumour cells leads to higher production of ROS and lipid metabolites, aiding ferroptosis development (Table 1; Figure 1). Tumours abundant in iron, like hepatocellular carcinoma (HCC) and breast cancer, or those rich in ROS like lung cancer, along with tumours with increased iron use and overload, demonstrate heightened sensitivity to ferroptosis (Ma et al., 2016).

Iron oxide nanoparticles, breaking down within the acidic tumour cell environment, release intracellular iron, leading to increased iron and ROS production, ultimately inducing ferroptosis and hindering tumour growth (Ma et al., 2017). Artemisinin prompts lung cancer cells to absorb and release substantial iron amounts, heightening their susceptibility to ferroptosis (Chen et al., 2020). In high-grade serous ovarian cancer (HGSOC), elevated iron intake and reduced expression of the iron efflux pump FPN result in excessive intracellular iron, further promoting ferroptosis onset (Basuli et al., 2017).

Thus, adjusting iron levels—enhancing iron intake, reducing storage, and restricting iron release—holds potential to promote ferroptosis and impede tumour growth.

## 4 Promotional effect of ferroptosis on tumour growth

**FIGURE 2:**
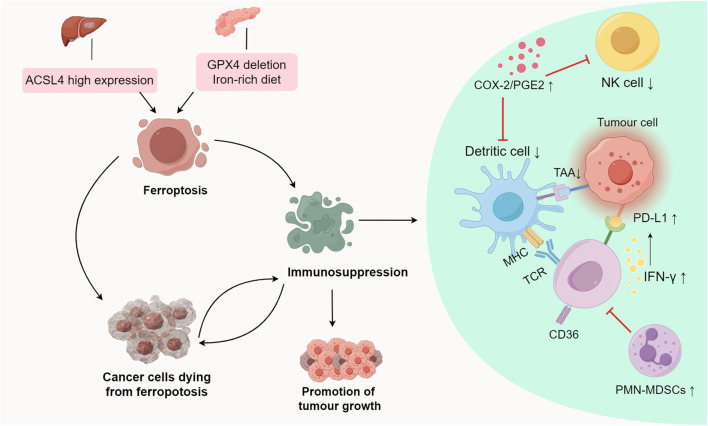
Promotional effect of ferroptosis on tumour growth.ACSL4-dependent ferroptosis promotes hepatocellular carcinoma development. A diet rich in iron or GPX4 depletion can induce ferroptosis, thereby promoting the development of pancreatic tumours. Ferroptosis inhibits antitumour immunity by triggering inflammation-related pathways, and influencing antigen presentation processes and the production of immunosuppressive molecules, thereby promoting tumour growth. Abbreviations: ACSL4, Acyl-coenzyme A synthetase long chain family member 4; GPX4, glutathione Peroxidase 4; PGE2, Prostaglandin E2; COX-2, cyclooxygenase-2; TAAs, tumour-associated antigens; IFN-γ, interferon-γ; PD-L1, Programmed cell death ligand 1; MHC, Major Histocompatibility Complex; PMN-MDSCs, polymorphonuclear myeloid derived suppressor cells.

Ferroptosis promotes tumour growth and progression by regulating metabolic pathways, triggering inflammation-associated immunosuppression, and cancer cells dying from ferroptosis to compromise antitumour immune responses (Figure 2; Table 2).

### 4.1 Ferroptosis promotes pancreatic tumour and hepatocellular carcinoma development and progression by regulating metabolic pathways

ACSL4, an enzyme involved in synthesizing phospholipids from PUFAs plays a crucial role in ferroptosis (Dixon et al., 2015; Yuan et al., 2016). In HCC, ACSL4 expression surpasses that in normal liver tissue, and hepatocyte ferroptosis relies on ACSL4, suggesting its involvement in HCC development (Ndiaye et al., 2020) (Figure 2). In a mature toxic injury model, intervention in ACSL4-dependent ferroptosis notably suppressed HCC progression, likely due to reduced fibrosis in the absence of ACSL4 (Tsurusaki et al., 2019; Li et al., 2020a; Luo et al., 2020; Qi et al., 2020) (Table 2).

Studies suggest that an iron-rich diet or Gpx4 depletion can induce ferroptosis in tumour cells, releasing 8-hydroxy-2′-deoxyguanosine (8-OHdG). This activates the TMEM173/STING-dependent DNA sensor pathway, thereby promoting pancreatic tumour development (Dai et al., 2020; Cooke et al., 2003) (Table 2). TMEM173 regulates inflammation and immune responses, associated with macrophage activation and migration triggered by 8-OHdG (Barber, 2015; Motwani et al., 2019). Tumour-associated macrophages (TAMs) influence early stages of pancreatic tumour formation, activating KRAS-driven PDACs (Mielgo and Schmid, 2013; Zhu et al., 2017). GPX4 deletion or a high-iron diet increases acinar-to-duct metaplasia, ductal lesions, stromal reactions, metastasis, as well as expression of Ki67 and a ferroptosis marker (prostaglandin-endoperoxide synthase 2, PTGS2) in the pancreas (Yang et al., 2014). These studies collectively suggest that ferroptosis contributes to promoting tumour growth.

### 4.2 Ferroptosis inhibits antitumour immunity by triggering inflammation-related pathways, thereby promoting tumour growth

#### 4.2.1 COX-2/PGE2 pathway

PTGS2, also known as cyclooxygenase-2 (COX-2), is a marker of ferroptosis (Yang et al., 2014). Lipopolysaccharide (LPS) induces lipid peroxidation and PTGS2 expression, which activates ferroptosis (Li et al., 2020b). Ferroptosis detrimentally influences tumour growth by specifically enhancing the COX-2/PGE2 pathway, leading to inflammation-associated immunosuppression (Tang et al., 2021; Li and Li, 2020; Yang et al., 2014) (Figure 2; Table 2). Prostaglandin E2 (PGE2), an inflammatory and immunosuppressive agent, undermines immune control mediated by conventional type 1 dendritic cells (cDC1s) and allows tumour cells to evade the immune system, resulting in immunotherapy resistance (Goodwin and Ceuppens, 1983; Wang and DuBois, 2015). Further studies reveal that PGE2 limits the infiltration of cDC1s into the tumour site by suppressing the chemokines CCL5 and XCL1, secreted by NK cells. Apart from impacting NK cell activity, PGE2 directly hampers cDC1s by reducing levels of tumour-recruited chemokine receptors (Böttcher et al., 2018; Zhang et al., 2022).

#### 4.2.2 IFN-γ-related immune resistance

Ferroptosis can induce immune response through ferroptotic tumour cells, exposing danger-associated molecular patterns (DAMPs) and releasing tumour-associated antigens (TAAs), which stimulate T cells to secrete Interferon-γ (IFN-γ) (Tang et al., 2019; Zhang et al., 2019). Though IFN-γ secretion by T cells induced by ferroptosis effectively eliminates tumour cells, it can also elevate programmed death ligand-1 (PD-L1) levels, triggering IFN-γ-related adaptive immune resistance (Dorand et al., 2016; Li et al., 2021) (Figure 2; Table 2). This phenomenon affects the antitumour efficiency of immune cells, contributing to tumour progression. PD-L1, an immunosuppressive molecule, is overexpressed in various cancers like gastric, kidney, pancreatic, bladder cancers, among others, and is linked to poor clinical prognosis (Ohigashi et al., 2005; Hamanishi et al., 2007; Nakanishi et al., 2007; Hou et al., 2014; Wang et al., 2016). Binding of PD-L1 to PD-1 depletes effector T cells, allowing tumour cells to evade the immune response, ultimately promoting tumour growth.

#### 4.2.3 CD36-mediated ferroptosis

The study noted notably elevated fatty acid levels in tumour tissues compared to normal skin or spleen tissues (Zhang et al., 2018). CD36, involved in DCs’ antigen presentation function, emerged as a T cell function regulator. The study showed that within the tumour microenvironment, CD8 (+) T cells uptake fatty acids via CD36, impairing their antitumour functionality by inducing ferroptosis and reducing cytotoxic cytokine production (Wang et al., 2020; Su et al., 2020) (Table 2). Effective blocking of CD36-mediated ferroptosis restored CD8 (+) T cells’ antitumour activity. Moreover, inhibiting CD36-mediated ferroptosis alongside immunotherapy notably enhanced the antitumour effects of CD8 (+) T cells (Wang et al., 2020).

### 4.3 Cancer cells dying from ferroptosis affects the antigen presentation process and produces immunosuppressive effects, thus compromising antitumour immune responses

Immunogenicity refers to antigens’ ability to provoke an immune response, involving immune effector molecule production, activation, proliferation, and differentiation (Aaes and Vandenabeele, 2021). Immunogenic cell death occurs when tumour-associated antigens (TAAs) are processed and presented on the surfaces of tumour cells and dendritic cells (DCs) (Legrand et al., 2019; Yatim et al., 2017). Research suggests that ferroptosis may possess immunomodulatory traits influencing neighbouring tumour cells’ response to immunogenic death (Blüml et al., 2005; Blüml et al., 2009). Observations indicate that cancer cells undergoing ferroptosis impede TAA processing and presentation. Co-culturing ferroptotic cancer cells with DCs revealed that these “initial” iron-depleted cells hinder DCs maturation, phagocytosis, and antigen-cross-presentation (Wiernicki et al., 2022) (Figure 2; Table 2). Consequently, ferroptosis negatively impacts antigen-presenting cells, influencing adaptive immune responses and antitumour immunity.

Pathologically activated neutrophils, termed polymorphonuclear myeloid-derived suppressor cells (PMN-MDSCs), negatively impact the regulation of antitumour immunity (Condamine et al., 2015; Wang et al., 2019b; Ostrand-Rosenberg et al., 2020). Their presence in cancer patients correlates with poor prognoses in immunotherapy (Zhou et al., 2018). Studies indicate that genes linked to PMN-MDSCs are enriched in the ferroptosis pathway, suggesting their susceptibility to ferroptosis (Zhang et al., 2020). Observations reveal that PMN-MDSCs can undergo ferroptosis within the tumour microenvironment. While ferroptosis reduces PMN-MDSC numbers, cancer cells undergoing ferroptosis release immunosuppressive molecules hindering T cells, reducing the effectiveness of antitumour therapy (Kim et al., 2022) (Table 2). Conversely, inhibiting ferroptosis decreases immunosuppressive activity, significantly impeding tumour growth. In immune-active mice, ferroptosis inhibition abolishes PMN-MDSCs’ suppressive activity on T cells and synergizes with immune checkpoint inhibitors to halt tumour progression (Kim et al., 2022). Hence, targeting ferroptosis in PMN-MDSCs holds promise as a future therapeutic avenue.

## 5 The role of ferroptosis in different cancer therapies, such as chemotherapy, immunotherapy, radiotherapy and reversal of tumour resistance

Currently, numerous studies corroborate the synergistic effect of ferroptosis combination therapy in bolstering the efficacy of antitumour treatment. However, evidence supporting the inhibitory effect of combination regimens remains relatively scarce. We’ve compiled studies showcasing the potential of combined ferroptosis therapy to heighten antitumour efficacy and surmount drug resistance (Table 3). Further pertinent research is warranted to build upon this foundation.

**TABLE 3:** Synergistic effect of ferroptosis combination therapy.

Category		Mode of functioning	Mechanism	*In vitro*/vivo	Model	Tumour type	References
Ferroptosis combination therapy	Chemotherapy	Erastin and cisplatin	ROS-mediated mechanism, diminish GSH, compromise GPX4 function, promote ferroptosis, augment the antitumour impact	*In vitro* and vivo	Human ovarian cancer cell lines (A2780, SKOV3, OVCA433, OVCAR5, OVCAR8 and HEY) and the human ovarian surface epithelial cell line (HOSEpiC); athymic BALB/c female nude mice; NSCLC cell lines (A549, NCIH358, NCIH460)	Ovarian cancer; NSCLC	Guo et al. (2018), Cheng et al. (2021)
		RSL3 and Paclitaxel	Downregulate SLC7A11, promote ferroptosis, retard tumour growth	*In vitro*	Human colorectal carcinoma cell line HCT116; HPSCC cells harboring mutant p53 (mtp53)	CRC, mutant P53 HPSCC	Lv et al. (2017), Ye et al. (2019)
	Radiation therapy	Radiation therapy	Generate ROS production, GSH depletion, ACSL4 upregulation, suppress SLC7A11 expression, enhance radiosensitivity	*In vitro*	H460, A549, H1299 cell lines	NSCLC	Azzam et al. (2012), Lang et al. (2019), Lei et al. (2020)
	Immunotherapy	CD8 (+) T cells release IFN-γ	Downregulate SLC7A11, reduce cystine uptake, induce ferroptosis	*In vitro* and vivo	B16 subcutaneous melanoma model; mice of HT-1080 cells	Melanoma; ovarian cancer	Wang et al. (2019a)
		BEBT-908	Increase MHC class I molecule expression, activate IFN-γ signaling pathway	*In vitro* and *in vivo*	NSCLC H2122 cells, CRC HCT116 cells; MC38 mouse colon adenocarcinoma cell line; female SCID mice with Daudi xenografts	CRC, human diffuse large B-cell lymphoma; lung cancer	Fan et al. (2021)
Reverse resistance		Anti-LCN2 monoclonal antibody (3D12B2)	Increase intracellular iron levels, decrease GPX4 expression, induce ferroptosis, overcome resistance	*In vitro* and *in vivo*	Colon cancer cell line HCT116; CD1 Nude mice	Colon cancer	Chaudhary et al. (2021)
		Vorinostat	Inhibit System Xc- and SLC7A11, stimulate ferroptosis in resistant cells	*In vitro*	EGFR mutant LUAD cell lines, HCC827, HCC4006, H1975, H1650, PC9, HCC4011 and H1993	EGFR-TKI resistant lung adenocarcinoma	Zhang et al. (2021)

ROS, reactive oxygen species; GPX4, glutathione Peroxidase 4; GSH, glutathione; NSCLC, non-small cell lung cancer; HPSCC, hypopharyngeal Squamous Carcinoma; ACSL4, Acyl-coenzyme A synthetase long chain family member 4; IFN-γ, Interferon-γ; LCN2, Lipocalin 2; CRC, colorectal cancer; LUAD, lung adenocarcinoma; EGFR, epidermal growth factor receptor; MHC, Major Histocompatibility Complex.

### 5.1 Ferroptosis combination with chemotherapy

Combining chemotherapeutic agents with ferroptosis inducers amplifies anticancer effects, as these drugs themselves can induce ferroptosis in tumour cells. For instance, the ferroptosis inducer erastin notably enhances cisplatin’s efficacy across various tumour types by antagonizing system Xc- or GPX4 functions (Yamaguchi et al., 2013; Yu et al., 2015). Studies illustrate that erastin combined with cisplatin impedes ovarian cancer progression via a ROS-mediated mechanism, augmenting cisplatin’s antitumour impact (Cheng et al., 2021) (Table 3). Moreover, cisplatin lowers GSH levels in tumour cells, compromising GPX4 function and triggering ferroptosis in NSCLC cells (Guo et al., 2018). Paclitaxel (PTX) reduces SLC7A11 expression, promoting ferroptosis and retarding colorectal carcinoma cell growth (Lv et al., 2017). The synergy of PTX with RSL3 induces ferroptosis in mutant p53 hypopharyngeal squamous carcinoma (Ye et al., 2019) (Figure 1; Table 3).

### 5.2 Ferroptosis combination with radiation therapy

Radiation therapy (RT) has been linked to inducing ferroptosis in tumours through diverse pathways (Lei et al., 2020), such as ROS production, GSH depletion, ACSL4 upregulation, and SLC7A11 inhibition (Azzam et al., 2012; Lei et al., 2020) (Figure 1). RT generates surplus ROS and triggers ACSL4 expression by breaking down cellular water, leading to PUFA peroxidation and ferroptosis (Ye et al., 2020). Additionally, RT can promote ferroptosis by suppressing SLC7A11 expression, thereby enhancing radiosensitivity (Lang et al., 2019). Ferroptosis inducers synergize with RT in tumour treatment. For instance, erastin and salazopyridine enhance NSCLC sensitivity to RT (Lei et al., 2020) (Figure 1; Table 3).

### 5.3 Ferroptosis combination with immunotherapy

The immune system wields significant influence over both tumour development and treatment. Recent research highlights ferroptosis as a factor impeding tumour growth by modulating the immune response (Wang et al., 2019a). Combining immune checkpoint inhibitors with ferroptosis inducers enhances immunotherapy efficacy (Jiang et al., 2021b). Ferroptosis, by recruiting and activating immune cells within the tumour environment, serves as a foundation for using ferroptosis inducers to augment immunotherapy. Ferroptotic tumour cells release DAMPs and trigger Major Histocompatibility Complex (MHC) class I molecule expression, activating T cells and macrophages (Wen et al., 2019). In the tumour microenvironment, CD8 (+) T cells produce IFN-γ, downregulating SLC7A11 expression, reducing cystine uptake, fostering lipid peroxide accumulation, and inducing ferroptosis in Melanoma and ovarian cancer cells (Wang et al., 2019a). The ferroptosis inducer BEBT-908 triggers ferroptosis, elevates MHC class I molecule expression, and activates the IFN-γ signalling pathway in Colorectal cancer (CRC), human diffuse large B-cell lymphoma, and lung cancer, bolstering the body’s immune response and exerting antitumour effects (Fan et al., 2021) (Figure 1; Table 3).

### 5.4 Ferroptosis reverses resistance to tumour therapy

Tumour drug resistance presents a formidable treatment challenge, often reinforced by tumour cells suppressing ferroptosis (Lu et al., 2017). Survival of drug-resistant cells often hinges on GPX4 (Hangauer et al., 2017). Inducing ferroptosis can reverse resistance to conventional chemotherapy, targeted therapy, and immunotherapy. In colon cancer, Lipocalin 2 (LCN2) overexpression leads to resistance to 5-fluorouracil. Targeted inhibition of LCN2 by anti-LCN2 monoclonal antibody (3D12B2) increases intracellular iron levels, decreases GPX4 expression, and induces ferroptosis in tumor cells, thereby overcoming resistance (Chaudhary et al., 2021) (Table 3). EGFR-mutated lung cancer cells, facing acquired drug resistance, exhibit heightened sensitivity to ferroptosis inducers. Vorinostat triggers ferroptosis in resistant cells by inhibiting System Xc- and SLC7A11 expression (Zhang et al., 2021) (Table 3). These findings underscore ferroptosis’ therapeutic potential in combatting drug resistance.

## 6 Future perspective and conclusion

We have summarized multiple studies delving into the interplay between ferroptosis and tumour growth, aiming to grasp their relationship comprehensively and provide insights for targeted therapeutic strategies. It’s vital to decipher how to counteract ferroptosis’ role in promoting tumour growth while harnessing its therapeutic potential. One study proposed RCH NPs, a self-amplifying nanomedicine, aiming to optimize therapeutic efficacy in tumours by addressing ferroptosis’ dual nature. RCH NPs displayed robust ferroptotic damage and bolstered the immune response, enhancing ferroptosis’ positive effects in inhibiting tumour growth. They also mitigated inflammation-linked immunosuppression and IFN-γ-associated adaptive immune resistance, countering ferroptosis’ negative impact on immunotherapy (Zhang et al., 2022). More research is anticipated to design effective therapies that balance ferroptosis’ dual effects on tumour growth.

Yet, ongoing research on ferroptosis remains in its early stages, leaving unanswered queries. Most studies investigating ferroptosis and its tumour association have relied on cellular and animal models, lacking validated clinical evidence. For instance, though targeting GPX4, a crucial component of the ferroptosis defense system, might theoretically restrain tumour growth, GPX4 is essential for life, with studies suggesting its loss heightens mortality rates in mice (Yant et al., 2003). Hence, it's crucial to ascertain the potential harm to normal tissue due to GPX4 inhibitors. Despite numerous potential targets linked to tumours and ferroptosis, it’s unclear which of these findings can transition into clinical investigations.

Furthermore, there’s a lack of research examining the effectiveness and safety of drugs intended to target ferroptosis. For instance, inhibiting SLC7A11 has shown potential in triggering tumour ferroptosis and reversing tumour resistance without observable impact on the development and survival of mice (Sato et al., 2005). However, this treatment may not be effective for tumours not reliant on the System Xc-. Ferroptosis is linked to the onset and progression of various diseases, extending beyond cancer to include degenerative conditions (Stockwell et al., 2017). Therefore, it’s crucial to develop tailored therapies inducing ferroptosis in tumours while avoiding systemic adverse reactions. Combination therapies involving ferroptosis inducers and RT have shown safety in preclinical studies, but there are indications that ferroptosis might also contribute to radiation-induced damage in normal tissues (Su et al., 2022). Hence, further research is necessary to understand the impact of ferroptosis inducers on normal tissues and identify the patient population most likely to benefit from these treatments.

Finally, there’s a notable absence of biomarkers available for assessing ferroptosis within the human body. Identifying suitable biomarkers would significantly aid in in vivo studies and clinical monitoring. Discovering predictive biomarkers capable of forecasting a tumour’s response to ferroptosis-inducing therapies is crucial for categorizing tumour patients and guiding subsequent antitumour interventions involving ferroptosis induction.

## Figures and Tables

**Table:** 

**GPX4**	glutathione peroxidase 4
**GSH**	glutathione
**PGE2**	Prostaglandin E2
**COX-2**	cyclooxygenase-2
**PD-L1**	programmed death ligand-1
**PUFAs**	Polyunsaturated fatty acids
**ROS**	reactive oxygen species
**ACSL4**	Acyl-coenzyme A synthetase long chain family member 4
**PUFA-PLs**	PUFA-containing phospholipids
**RT**	radiation therapy
**LPCAT3**	Lysophosphatidylcholine acyltransferase 3
**LOXs**	lipoxygenase
**PORs**	cytochrome P450 oxidoreductase
**ACSL3**	Acyl-coenzyme A synthetase long chain family member 3
**MUFA-PL**	Monounsaturated fatty acids-phospholipids
**FDFT1/SQS**	farnesyl-diphosphate farnesyltransferase 1
**CRC**	Colorectal cancer
**ELOVL5**	elongation of very long-chain fatty acid protein 5
**FADS1**	fatty acid desaturase 1
**PDACs**	pancreatic ductal adenocarcinomas
**HNC**	head and neck cancer
**RCC**	renal cell carcinoma
**HCC**	hepatocellular carcinoma
**NSCLC**	non-small cell lung cancer
**TFR**	transferrin
**DMT1**	divalent metal ion transporter protein-1
**HGSOC**	high-grade serous ovarian cancer
**TAMs**	Tumour-associated macrophages
**PTGS2**	prostaglandin-endoperoxide synthase 2
**cDC1s**	conventional type 1 dendritic cells
**DCs**	dendritic cells
**DAMPs**	damage-related molecular patterns
**PMN-MDSCs**	polymorphonuclear myeloid derived suppressor cells
**IFN-γ**	Interferon-γ
**lncRNA**	long non-coding RNA
**LCN2**	Lipocalin 2
**MHC**	Major Histocompatibility Complex
**8-OHdG**	8-hydroxy-2′-deoxyguanosine
**PTX**	paclitaxel
**TAAs**	tumour-associated antigens
**PLA2**	Phospholipase A2
**LUAD**	lung adenocarcinoma
**FPN**	ferroportin
**LPS**	Lipopolysaccharide
